# Tumour immune infiltration is independent of peripheral circulation of white blood cells in glioblastoma

**DOI:** 10.1038/s41598-025-16260-6

**Published:** 2025-08-26

**Authors:** Anabel García-Heredia, Luna Guerra-Núñez, Marianela Iriarte-Gahete, Pablo Espinosa-Lara, Luis M. Valor

**Affiliations:** 1 Research Laboratory, Dr. Balmis General University Hospital, Alicante Institute for Health and Biomedical Research (ISABIAL), Alicante, 03010 Spain; 2 Department of Haematology, Immunology and Genetics, Puerta del Mar University Hospital, Cadiz, 11009 Spain; 3 Department of Dermatology, Infanta Elena Hospital, Huelva, 21080 Spain; 4 Institute of Research, Development, and Innovation in Healthcare Biotechnology in Elche (IDiBE), Miguel Hernández University, Elche, 03202 Spain; 5 Research Laboratory, Dr. Balmis General University Hospital, ISABIAL, 6th floor Diagnostics Building, Av. Pintor Baeza 12, Alicante, 03010 Spain; 6 Present Address: Immunology Service, Unit of Clinical Laboratories, Institute of Biomedicine of Seville, IBiS/Virgen del Rocío University Hospital, CSIC/University of Seville, Seville, 41013 Spain

**Keywords:** Glioma, NLR, MLR, Neutrophil, Macrophage, Microenvironment, Blood, Transcriptomics, CNS cancer, Molecular medicine, Biomarkers

## Abstract

Glioblastomas are the most prevalent and malignant primary cancers in the brain. Given the promising prospects of immunotherapeutic approaches, there is increasing interest in obtaining precise knowledge of the immunologic status of the tumour microenvironment for each individual. We explored the feasibility of inferring this tumour immune component in a minimally invasive manner prior to any clinical intervention, taking advantage of the preoperative immune cell counts that can be easily obtained from the clinical records of the patients. The neutrophil-to-lymphocyte ratio (NLR) is the most extensive measure calculated from complete counts of peripheral blood. Despite there was an increase in the NLR in high grade tumours of our cohorts of study, we did not find evidence of any correlation between the NLR and different degrees of tumour immune infiltration in glioblastoma. The same negative result was obtained with the monocyte-to-lymphocyte ratio (MLR). In addition, glioblastomas associated with extreme values of peripheral NLR did not exhibit substantial gene expression differences that could be linked to distinct tumourigenic properties. Overall, these results suggest that peripheral immune cell ratios cannot be used to reliably infer the immune microenvironment within the tumour, underscoring the complexity of using peripheral markers to assess local tumour immunity.

**Supplementary Information:**

The online version contains supplementary material available at 10.1038/s41598-025-16260-6.

## Introduction

Primary brain cancer is highly heterogeneous and can be especially aggressive as in the case of glioblastomas. In recent years, the tumour microenvironment has received increasing attention as a more profound understanding may pave the way for the successful application of immunotherapies^1–3^. In this microenvironment, neoplastic and nontumoural cells establish dynamic and complex interrelations that modulate the progression and therapeutic response of the tumour. For example, a high proportion of tumour-associated macrophages (TAMs) has been associated with poor prognosis in several cancer types, mainly because of their capability to promote angiogenesis, invasion and supression of the antitumour immunity^4–7^. TAMs have been the main focus of the tumour microenvironment because they constitute the predominant immune cell population, accounting for ~ 40% of the cellular composition of tumours. However, other cell types can also exert either immunosuppressive or antitumour effects, as exemplified by tumour-associated neutrophils (TANs)^8–11^. In any case, infiltrating immune cell types tend to share the same tumoural niche^12^; therefore, it is plausible that the modulation of any of these cellular types may influence the activity of the remaining subpopulations within the niche.

In contrast to neutrophils in the brain, this cell type is the most abundant circulating white cells in blood (40−60% compared with 2−8% of monocytes). As part of the innate immune system, neutrophils are among the first responders to inflammation. Capable of phagocytosis, degranulation and the formation of neutrophil extracellular traps to combat infection^13^, circulating neutrophils have been proposed as inflammatory biomarkers of neurological conditions, serving as accessible proxies of elevated inflammation during the early steps of neuropathological processes^14–17^. In this regard, the neutrophil-to-lymphocyte ratio (NLR) has become the preferred measure over other parameters such as the platelet-to-lymphocyte ratio (PLR), systemic immune-inflammation index (SII) and monocyte-to-lymphocyte ratio (MLR). In the particular case of glioblastoma, NLR has been linked to poor prognosis^18–24^ although this association is still under debate^25^. Keeping in mind that complete blood count is part of routine clinical practice prior to brain surgery and can be easily performed at any stage of the disease, peripheral immune profiling could provide rapid information about the status of the patient and predict future outcomes in a cost-effective manner.

Peripheral immune profiles may be used as surrogates for otherwise inaccessible cancer processes. For this reason, we hypothesized that blood count data may be correlated with tumour immune infiltration, offering affordable options for patient monitoring, outcome prediction, and eligibility criteria for immunotherapy. To explore this hypothesis, we correlated for the first time the presence of immune cells in brain tumours and peripheral blood from the same patients with glioblastoma.

## Materials and methods

### RNA-seq datasets

Glioblastoma-related datasets from The Cancer Genome Atlas (TCGA) were retrieved from the “GBM project” through ‘TCGAbiolinks’^26^ from which 161 IDH-wildtype tumours were further selected for subsequent analyses, following the updated 2021 WHO recommendations^27^. In addition, we used two independent cohorts consisting of 23 glioblastomas derived from fresh-frozen tumours resected at the Hospital Universitario Puerta del Mar, namely, Cohort #1 (reported in previous publications^28,29^ available in the GEO repository under accession number GSE185861, with a median (IQR) age = 58 (49–66) and a proportion of 56.5% men and 43.5% women), and 113 glioblastomas from FFPE-derived biomaterials of the Hospital General Universitario Dr. Balmis, namely, Cohort #2 (processed as described previously^30^ available under accession number GSE272042, with a median (IQR) age = 62 (53–71) and a proportion of 58.4% men and 41.6% women).

### Compilation of clinical data

Presurgical complete blood counts from extended versions of our cohorts that included additional glioblastomas and other primary brain cancers were examined to calculate the following ratios: neutrophil-to-lymphocyte ratio (NLR) and monocyte-to-lymphocyte ratio (MLR). The general characteristics of these patients were as follows: Extended Cohort #1, median (IQR) age = 54 (44.5–63.5) and proportion of 55.4% men and 44.6% women; Extended Cohort #2, median (IQR) of age = 56 (40–65) and a proportion of 61% men and 39% women.

### Bioinformatics and statistical analysis

The same processing pipeline was consistently applied to all datasets used in the study, ensuring uniformity in data handling and analysis across the different cohorts. The resulting FASTQ files were aligned to the GRCh38 human genome (GENCODE 43/Ensembl 109) and processed with ‘Salmon’ to quantify the reads associated with each transcript^31^. The output provided abundance counts per transcript, which were then converted to gene-level counts using the Bioconductor package ‘tximport’^32^.

The relative proportions of immune, stromal, and neoplastic cell populations were estimated from bulk RNA-sequencing expression profiles (transcripts per million or TPM) using the ‘GBMDeconvoluteR’ software^33^. To separate the population of cells into two clusters, the k-means clustering algorithm from the Waikato Environment for Knowledge Analysis (Weka) (v3.8.6) was employed^34^.

Statistical analysis was carried out using the R environment (v4.4.1) that included correlation analyses and differences between two (Mann-Whitney *U* tests) or more groups (Kruskal-Wallis tests). Survival analysis was performed via ‘survival’ (v3.7.0) and ‘survminer’ (v0.4.9) packages to plot the Kaplan–Meier curves and calculate log-rank *p*-values.‘DESeq2’ package was used for differential gene expression analysis^35^. Overrepresentation analysis of Gene Ontology (GO) terms of biological processes was performed on the differentially expressed genes (DEGs) using the DAVID Knowledgebase v2024q2^36^. Data visualizations were created with the ‘ggplot2’ and ‘EnhancedVolcano’ packages.

### Ethics approval

The study was conducted according to the guidelines of the Declaration of Helsinki and approved by the local ethics committees (Comité de Ética de la Investigación de Cádiz, ref. 38/19; Comité de Ética de Investigación Clínica con medicamentos del Hospital General Universitario Dr. Balmis, ref. PI2022-128) according to the national and regional laws and regulations concerning biomedical research on human samples, personal data protection and the use of biobank services. For Cohort #2, samples and clinical data were provided by the BioBank of ISABIAL which is adhered to the Spanish National Biobanks Network and integrated in the Valencian Biobanking Network, following standard operating procedures under the approval of the local ethics committee. Informed consent was obtained from participants with the exception of those cases waived by the aforementioned ethics committees.

## Results

### Glioblastoma tumours with high proportions of immune cells tend to be especially aggressive

**Fig. 1:**
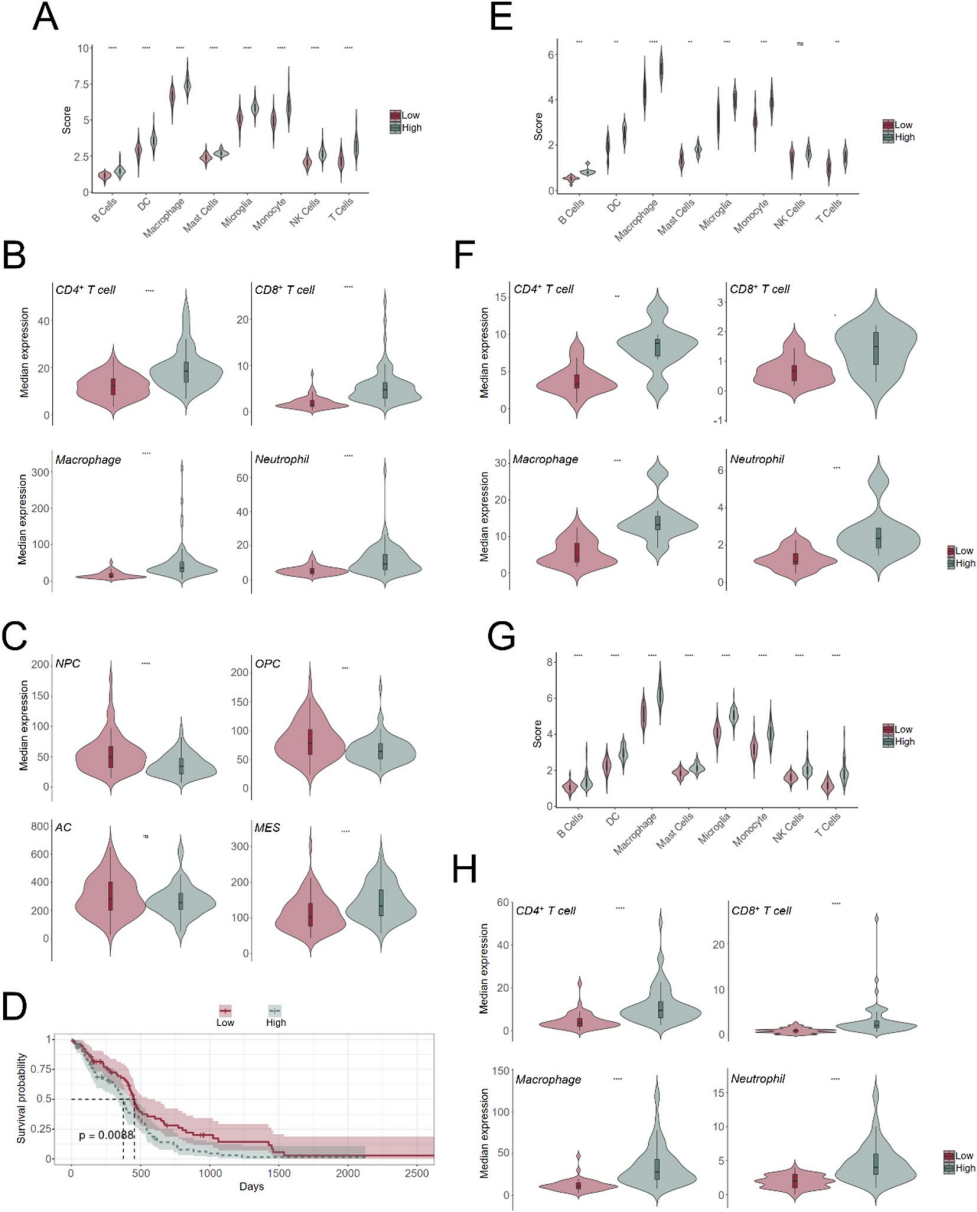
Glioblastomas can be classified in two main clusters based on their tumour immune cell content. (**A**,**B**,**C**,**D**), TCGA glioblastomas. (**A**), Violin plots of the scores for each immune cell type contained in the “GBMDeconvoluteR” tool in clusters “Low” (*n* = 83) and “High” (*n* = 78). (**B**), Violin plots of the gene expression levels of selected immune cell markers for CD4^+^ and CD8^+^ T-cells, macrophages and neutrophils in clusters “Low” and “High”. (**C**), Same as *B* with neoplastic cell subtypes: NPC, neural progenitor cell-like; OPC, oligodendrocyte progenitor cell-like; AC, astrocytic-like; MES, mesenchymal-like. (**D**), Kaplan-Meier curve for clusters “Low” and “High” indicating the log-rank *p*-value. (**E**,**F**), Glioblastomas of Cohort #1. (**E**), Same as *A*, in which clusters “Low” and “High” were composed of *n* = 15 and 8, respectively. (**F**), Same as *B*. (**G**,**H**), Glioblastomas of Cohort #2. (**G**), Same as *A*, in which clusters “Low” and “High” were composed of *n* = 48 and 65, respectively. (**H**), Same as *B*. In all panels: ns, not significant; *, *p*-value < 0.05; **, *p*-value < 0.01; ***, *p*-value < 0.001; ****, *p*-value < 0.0001 (“Low” vs. “High”), Mann-Whitney *U*-test.

We used the “GBMDeconvoluteR” tool to infer the proportions of different cell types in the transcriptional profiles from the bulk RNA-seq datasets. This tool takes advantage of single cell-type markers for neoplastic cells (astrocyte-like, oligodendrocyte-like and neuronal progenitor-like and mesenchymal) and immune cells (microglia, tumor associated macrophages, monocytes, B, T, NK, mast cells and dendritic cells or DCs) to provide scores indicating the proportion of each of these cell types within each glioma^33^. Using this tool, we classified glioblastomas from the TCGA database according to the immune cell subtype scores: two clusters were sufficient to divide the tumours into “High” and “Low” subtypes, reflecting different global proportions of immune cells (Fig. 1A). To validate these clusters we used markers retrieved from independent studies^37,38^ for CD4^+^ T cells (*CD3D*, *CD3G*,* CD4*, *IL7R*, *LDHB*, *LTB*, *MAL*, *TMSB10*, *TPT1*, *TRAC*), CD8^+^ T cells (*CD3D*, *CD3E*, *CD3G*, *CD8A*, *CD8B*, *CTSW*, *HCST*, *LINC02446*, *TMSB10*, *TRAC*), macrophages (*C1QA*, *C1QB*, *C1QC*, *CCL18*, *CD163*, *CD163L1*, *CD5L*, *COLEC12*, *F13A1*, *FABP3*, *FABP4*, *MRC1*, *MS4A6A*, *SLC1A3*, *SPIC*, *STAB1*, *TLR2*) and neutrophils, a cell subpopulation that was missing in “GBMDeconvoluteR” (*CD274*, *CSF3R*, *CXCL8*, *CXCR1*, *CXCR2*, *FCGR3B*, *FFAR2*, *FOSL1*, *FUT4*, *GOS2*, *MMP9*, *PTPRC*, *S100A8*). We confirmed that the median expression of these markers was significantly larger in the “High” cluster than in the “Low” cluster (Fig. 1B). Notably, both clusters differed in the proportions of neoplastic cell subtypes, with the mesenchymal signature being overexpressed in the “High” cluster whereas the signatures linked to cancer cells resembling neuronal (NPC) and oligodendrocyte precursor cells (OPC) were significantly enriched in the “Low” cluster (Fig. 1C). The overall survival of the “Low” tumours was significantly larger than the overall survival of the “High” tumours (Fig. 1D), which was in agreement with the reported association between profuse immune cell infiltration and poor prognosis^7,39^ and with the more prominent presence of the mesenchymal signature which is linked to glioma aggressivity and worse prognosis^40,41^. Next, we conducted the same deconvolution analyses in our own independent datasets derived from fresh-frozen (Cohort #1, Fig. 1E-F) and FFPE-derived (Cohort #2, Fig. 1G-H) glioblastomas, which demonstrated that these cohorts could also be classified into “High” and “Low” categories according to the expression of immune cell markers. Although not significant, we observed a trend towards longer survival for those tumours with lower immunogenic load in Cohort #2 (Supplementary Fig. S1).

To better characterize the two identified groups of glioblastomas, we estimated the proportion of sexes and the averaged age of diagnosis, since the prevalence is higher in men and age is a well established risk factor^42^. Despite observing a trend towards men and advance age for “High” glioblastomas in all examined cohorts, this was not statistically different (Supplementary Fig. S2), suggesting that these factors were not sufficiently associated with tumoural immune content.

### The neutrophil-to-lymphocyte ratio is increased in the aggressive glioblastomas but it is not related to tumour immune infiltration

**Fig. 2:**
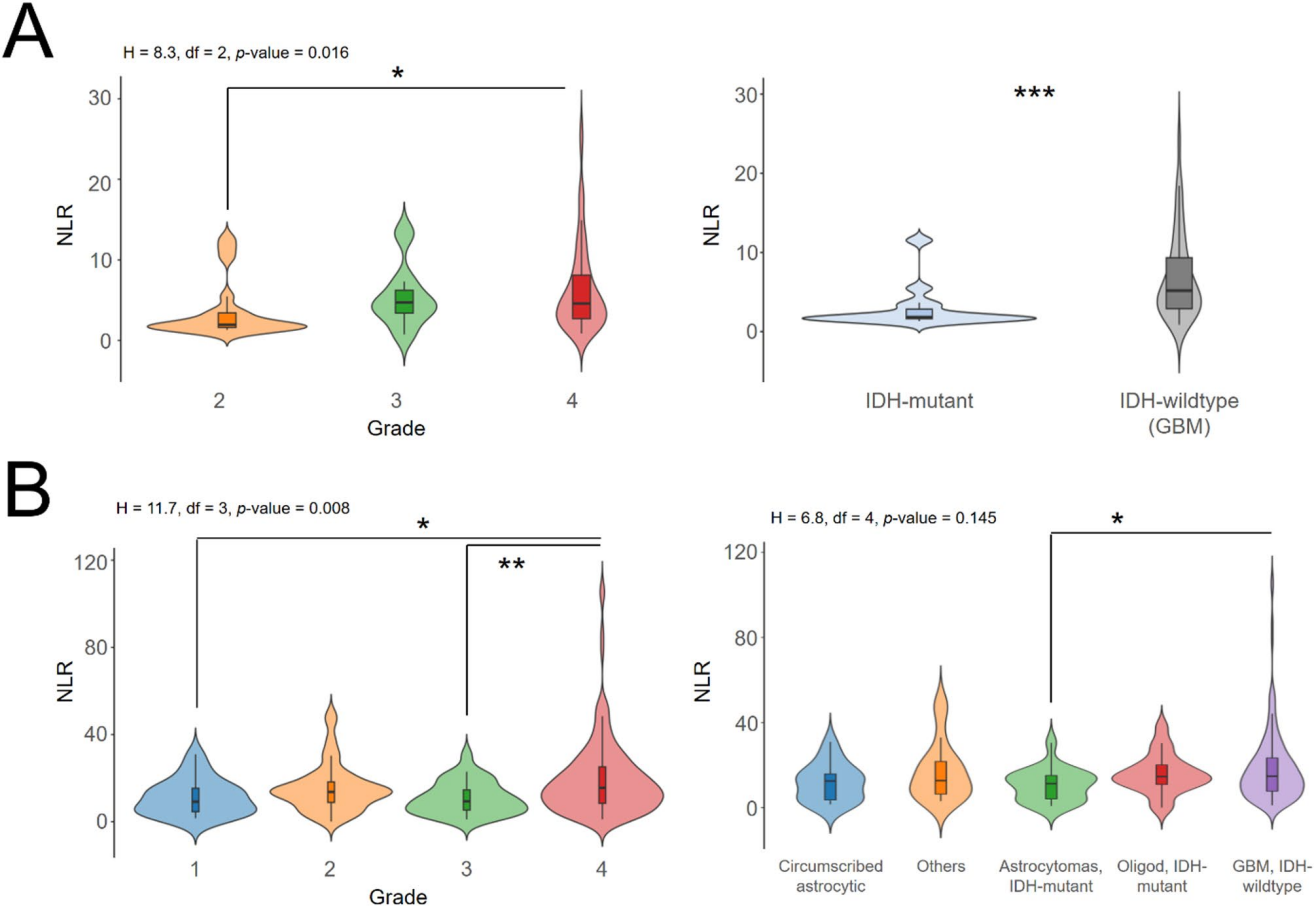
Neutrophil-to-Lymphocyte Ratios is increased in the most aggressive glioma tumours. (**A**), NLRs associated to primary brain cancers of Extended Cohort #1, according to histological grade (left panel): *n* = 20, grade 2; *n* = 7, grade 3; *n* = 65, grade 4. NLRs associated to primary brain cancers of Extended Cohort #1, according to the IDH1/2 mutation (right panel); *n* = 18, IDHmutant; *n* = 52, IDHwildtype. (**B**), NLRs associated to primary brain cancers of Extended Cohort #2, according to histological grade (left panel): *n* = 19, grade 1; *n* = 43, grade 2; *n* = 29, grade 3; *n* = 148, grade 4. NLRs associated to primary brain cancers of Extended Cohort #2, according to 2021 WHO diagnosis (right panel): *n* = 16, circumscribed astrocytic gliomas (pilocytic astrocytoma, pleomorphic xanthoastrocytoma); *n* = 19, astrocytoma IDHmutant; *n* = 19, oligodendroglioma IDHmutant and 1p/19q codeleted; *n* = 173, glioblastoma IDHwildtype; *n* = 18, others (ganglioglioma, ependymoma). ns, not significant; *, *p*-value < 0.05; **, *p*-value < 0.01; ***, *p*-value < 0.001; Kruskal-Wallis test followed by post-hoc Mann-Whitney’s *U*-test.

**Fig. 3:**
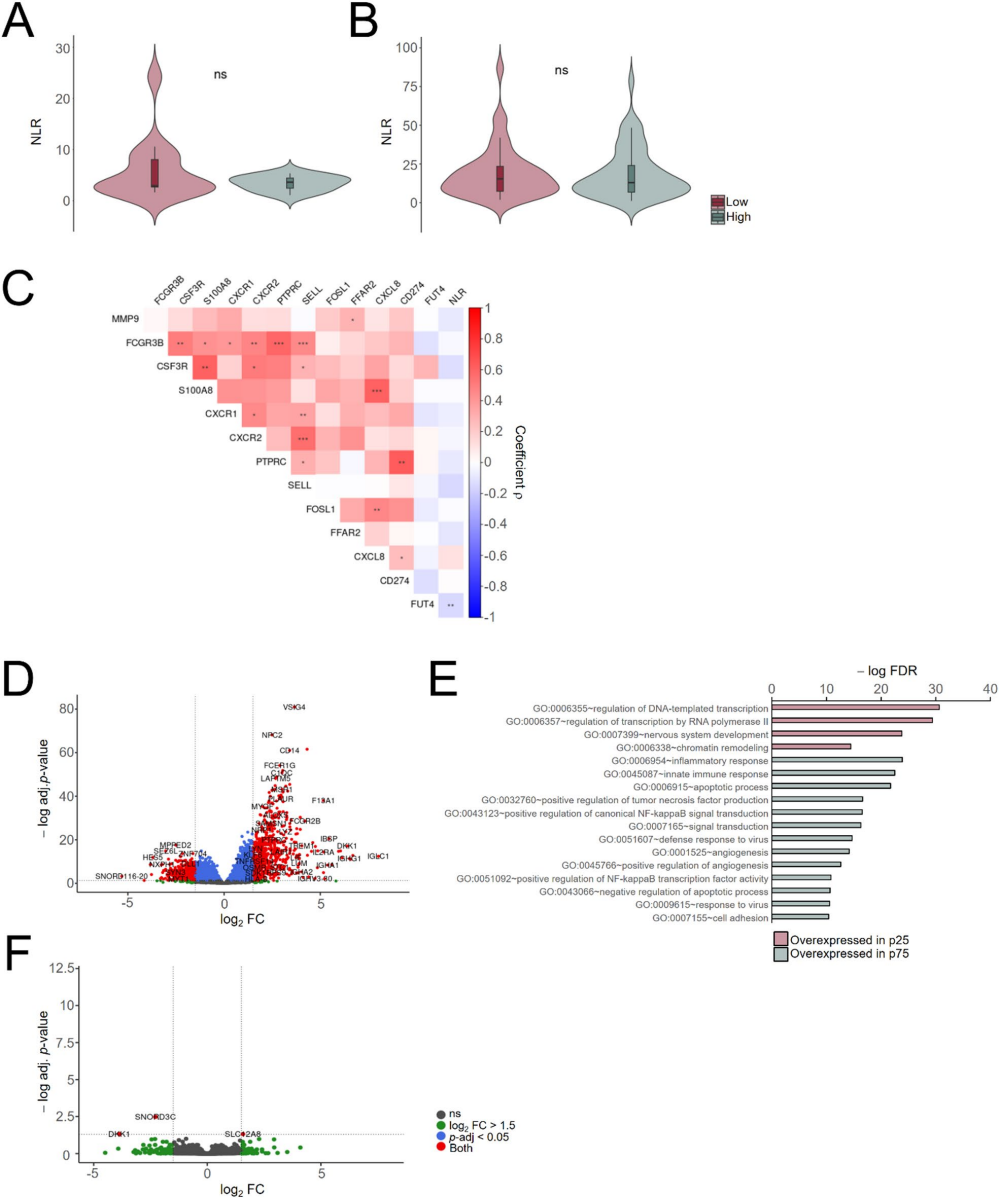
Neutrophile-to-Lymphocyte Ratios are not linked to tumour immune infiltration in glioblastoma. (**A**,** B**), Violin plots of NLRs in clusters “Low” and “High” of Cohort #1 (**A**) and #2 (**B**). (**C**), Spearman correlation coefficients (ρ) between neutrophil markers and NLR in Cohort #2. ns, not significant, *, *p*-value < 0.05; **, *p*-value < 0.01; ***, *p*-value < 0.001; ****, *p*-value < 0.0001. *D*, Volcano plot showing the results of the differential expression analysis between glioblastomas with the most extreme macrophage scores (25th vs. 75th percentiles). (**E**), Enrichment analysis of GO terms related with Biological Processes in the differentially expressed genes (adj. *p*-value < 0.05) depicted in (**D**). Only GO terms with FDR < 10^−10^ are shown (see Supplementary Material for complete results). (**F**), Volcano plot showing the results of the differential expression analysis between glioblastomas with the most extreme NLR values (percentiles 25th vs 75th); out of the plot there are the following genes: *RNA5S16* (log_2_ fold change= −25.2,−log adj. *p*-value = 5.2) and *RNA5S12* (log_2_ fold change = 25.0,−log adj. *p*-value = 5.2).

Taking advantage of available complete cell count analyses that are routinely performed prior to brain surgery, we calculated the NLR in our cohorts as this parameter has been extensively used for its potential prognostic value. NLR values were generally increased in high grade gliomas related to low grade gliomas in both Cohort #1 and Cohort #2 (Fig. 2A-B, left panels), especially in glioblastomas, which exhibited the most extreme values (Fig. 2A-B, right panels), suggesting a relationship between the NLR and glioma aggressivity. Therefore, we investigated whether there was a correlation between the NLR and the presence of intratumoural infiltrated immune cells. However, the “High” and “Low” clusters of glioblastomas did not differ in the overall NLR values in our two independent cohorts (Fig. 3A, B). In fact, no correlation was observed with the gene expression profile of the neutrophil markers found within tumours and the associated NLR from the same patients in the larger Cohort #2, whereas most of these markers expectedly correlated with each other (Fig. 3C). Lastly, we calculated the tumoural NLR values (median of neutrophil markers expression/median of CD4^+^ T markers expression, as the latter is the most abundant lymphocyte cell type in brain and glioblastomas) that were correlated with peripheral NLR values, but again this was not significant (Kendall’s coefficient = −0.08, *p*-value = 0.464).

### The neutrophil-to-lymphocyte ratio does not provide a distinctive tumour expression profile

Although the NLR and tumour immune infiltration were not correlated, they might still be useful for determining distinct biological properties within glioblastomas separately. To this end, we performed two parallel differential expression analyses: one between the glioblastomas with the highest and lowest scores of infiltrating macrophages (as the most notorious example of tumour immune cells), and another with the highest and lowest NLR values; in both cases, we performed pairwise comparisons between those samples showing with the most extreme values corresponding to the 25th and 75th percentiles (*n* = 23–24 per condition). In the first case, we retrieved 2610 and 3405 genes that were overexpressed in the most “High” and most “Low” tumours, respectively (adj. *p*-value < 0.05) (Fig. 3D). As expected, the Gene Ontology analysis confirmed the enrichment of genes related to inflammation in the most “High” tumours, whereas genes related to NPC (“nervous system development”) were overexpressed in the most “Low” tumours (Fig. 3E). These results reflected the overall changes in the cellular composition of the tumour due to immune infiltration, together with indications of altered functions such as angiogenesis, NF-κB-dependent signalling pathways, apoptosis and cell adhesion (“High”) as well as transcriptional regulation (“Low”) (Fig. 3E), among many others (Supplementary Fig. S3). In contrast, only 5 genes were differentially expressed (*DKK1*,* RNA5S12*, *RNA5S16*, *SLC12A8*,* SNORD3C*) between tumours with the highest and lowest NLR values (Fig. 3F), therefore variations of the peripheral NLR values were unable to be used to determine different subtypes of tumoural processes with potential clinical relevance.

### Circulating monocytes are not peripheral correlates of the tumoural immune load

**Fig. 4:**
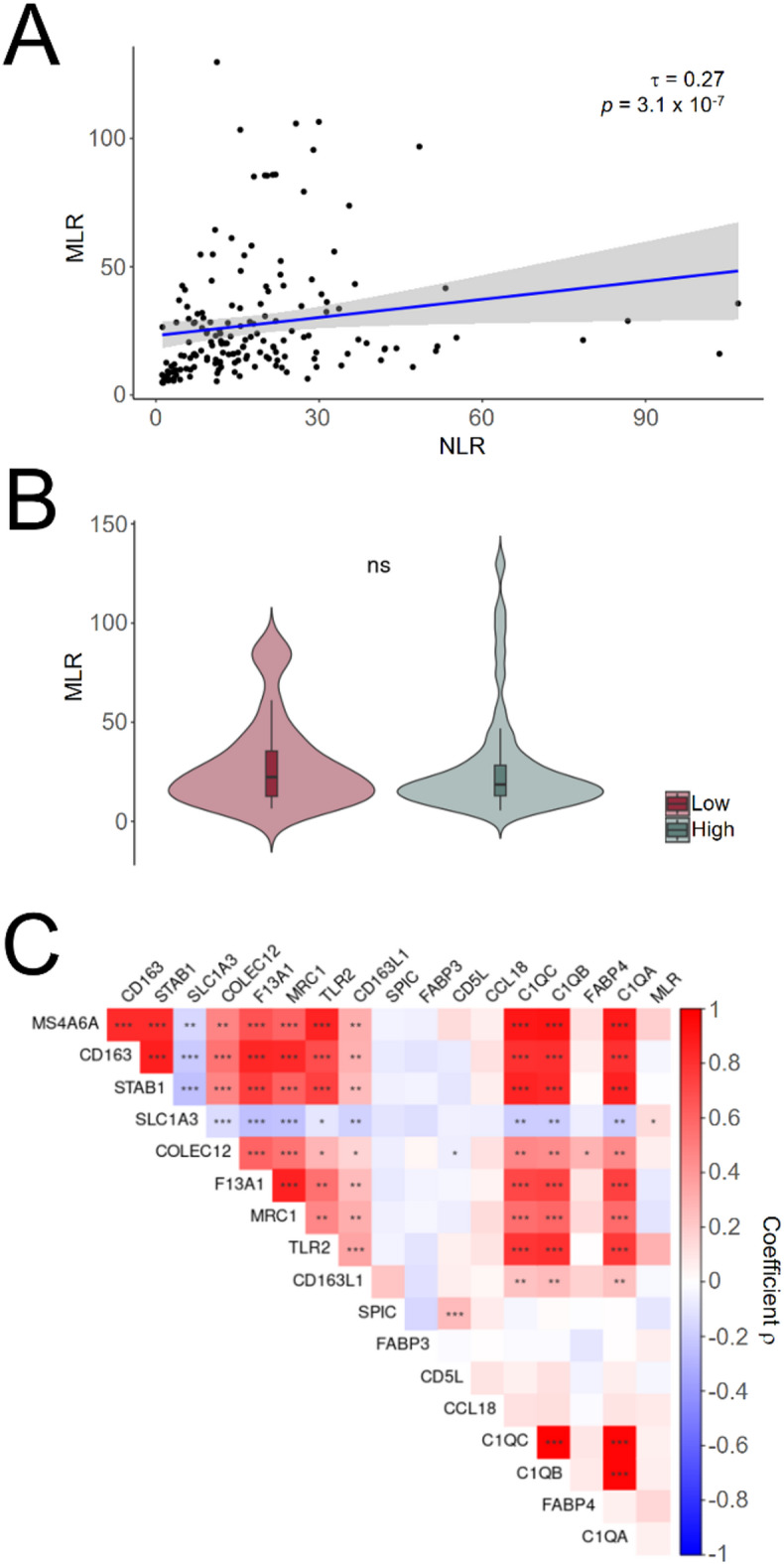
Monocyte-to-Lymphocyte Ratios are not linked to tumour immune infiltration in glioblastoma. (**A**), Kendall’s rank correlation between MLR and NLR in Extended Cohort #2. (**B**), Violin plot of MLRs in clusters “Low” and “High” of Cohort #2. (**C**), Spearman correlation coefficients (ρ) between macrophage markers and MLR in Cohort #2. ns, not significant; *, *p*-value < 0.05; **, *p*-value < 0.01; ***, *p*-value < 0.001; ****, *p*-value < 0.0001.

Our previous results indicated that the NLR is not informative regarding the immune component of the tumoural microenvironment. We investigated an alternative peripheral measure, the MLR, considering that the bloodstream supplies the circulating monocytes that infiltrate and differentiate into the most abundant cell type in brain tumours, TAMs. Although both the NLR and MLR were significantly correlated, Kendall’s coefficient was moderate (Fig. 4A), indicating that further exploration of the latter parameter was justified. However, the “High” and “Low” clusters did not differ in terms of the MLR (Fig. 4B), resembling the results retrieved with the NLR. When investigating correlations between the MLR and the tumoural expression of specific macrophage markers, only *SLC1A3* was significant; however, this marker also accounts for the expression of the astrocytic glutamate transporter GLAST-1, probably explaining the negative correlations observed with the expression of other myeloid markers (Fig. 4C). The correlation of MLR between blood and tumours (median of macrophage markers expression/median of CD4^+^ T markers expression) was also not significant (Kendall’s coefficient = −0.17, *p*-value = 0.126).

## Discussion

In this study, we discarded the use of the NLR and MLR from presurgical blood cell counts to infer immune infiltration in brain tumour as we did not find any association. However, the NLR varied across histological grades and IDH mutation status, indicating that this peripheral measure has prognostic value across broad glioma subtypes, although we did not find that it is applicable within glioblastomas. The uncoupling between peripheral neutrophil ratios and expression levels of tumoural neutrophil markers can be explained by the influence of the tumoural microenvironment on TANs, as these neutrophils undergo phenotypic changes, acquiring a prolonged lifespan along with immunosupressive and proangiogenic properties, compared to their peripheral counterparts^37^, therefore evolving as separate cell subpopulations during the tumour development. Thus, our results support the idea that peripheral immune profiles might fail to capture the intricate dynamics of the local immune landscape within gliomas, where the interactions between different immune cell populations may obscure the significance of peripheral markers. Thus, the tumour microenvironment may require more specific biomarkers or more integrated analyses to better reflect its immunological state.

Notably, glioblastomas associated with significant peripheral NLR differences did not exhibit substantial transcriptional differences, probably due to the complex convergence of confounding factors that impedes a better understanding of the potential association between the NLR and poor prognosis in brain cancer patients. Meanwhile sex was not relevant for peripheral cell counts, we observed very mild correlations with age of diagnosis (Supplementary Fig. S2) in agreement with reported aging dynamics for circulating neutrophil and monocyte counts^43,44^. The use of corticosteroids (e.g., dexamethasone) can have a more profound influence on peripheral blood counts, generally by increasing neutrophil numbers among other blood cells variations, deeply affecting presurgical NLR values^45–48^. Alongside medication, other confounding factors, such as obesity, stress, smoking, metabolic or genetic conditions among others, may also mask the individual’s reaction to the presence of the tumour^49^ and may explain altogether the controversy regarding whether high NLR values can be used as a predictor of poor prognosis in various cancers including glioblastoma^50–54^.

Although our findings indicate that the NLR (and the MLR as well) is not predictive of immune infiltration in glioblastoma, the development of combined approaches that integrate peripheral biomarkers with molecular analyses of the tumour microenvironment could provide a more comprehensive assessment of prognosis and treatment response. New combinations that involve different factors (e.g., medication history and habits) are needed to enhance these predictive models and should also include longitudinal analysis of clinical parameters during cancer progression. Future studies should focus on analysing cytokine profiles and other immune mediators in serum and tumour tissue, thanks to advanced techniques such as immunophenotyping or proteomics analysis, to better characterize the immune microenvironment in glioblastoma and its relationship with tumour progression.

### Limitation of the study

Our work did not consider the immunomodulatory effects of steroids, prominently dexamethasone, which are widely used in a presurgical stage to alleviate the symptoms generally linked to brain tumours (headaches, vomiting and others) by reducing focal edemas and intracranial pressure and by preserving the integrity of the brain-blood barrier, thanks to their anti-inflammatory properties^55^. This type of treatment is applied to the majority of patients with brain tumours (as in the case of the glioblastomas analyzed here) in a variety of regimes that, as already discussed, may be one of the most prominent confounding factors in the peripheral values obtained in the present study. Further research will determine the precise contribution of dexamethasone treatment to the immunological-based parameters associated with glioblastoma in our cohorts.

## Supplementary Information

Below is the link to the electronic supplementary material.

Supplementary Material 1

## Data Availability

The RNA-seq data can be downloaded from the Gene Expression Omnibuos (GEO) database under the accession numbers GSE185861 and GSE272042.
