# Supplementary material for: Tumour immune infiltration is independent of peripheral circulation of white blood cells in glioblastoma
Source: Sci Rep. 2025 Aug 26;15:31344. doi: 10.1038/s41598-025-16260-6 (PMC12381165; doi:10.1038/s41598-025-16260-6)
Supplement: Supplementary file 1 — Supplementary Material 1 [file 41598_2025_16260_MOESM1_ESM.pdf]

Supplementary Figure S1. Overall survival analysis of glioblastomas with different tumoural immune content from Cohort #2

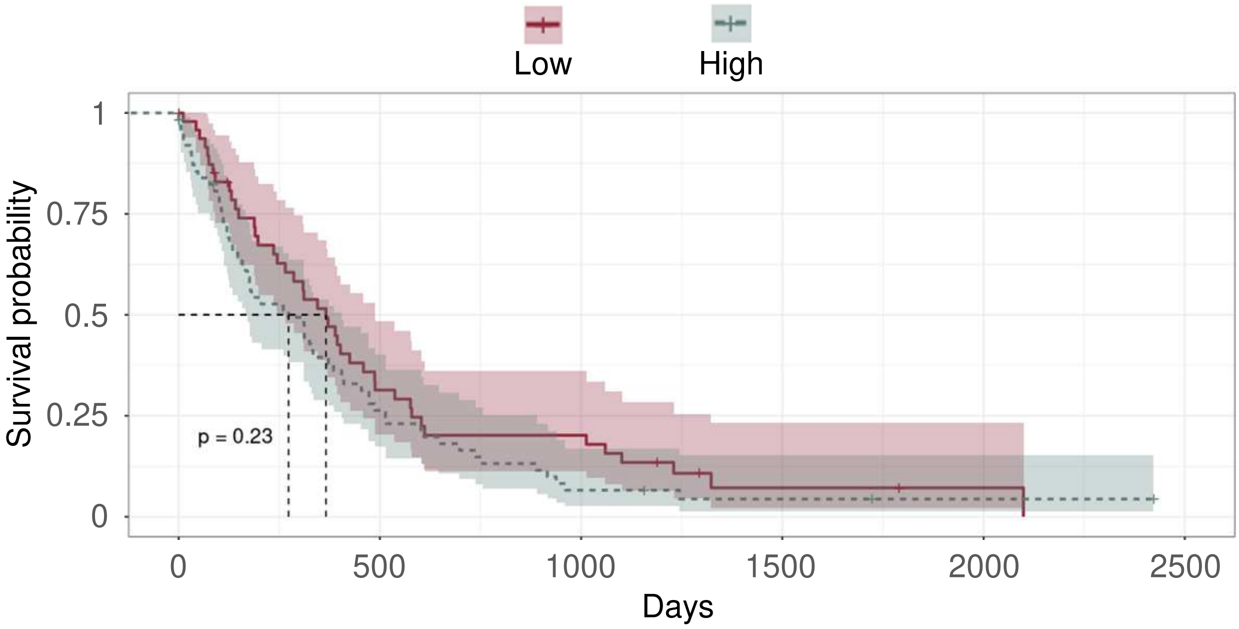

Kaplan-Meier curve for clusters “Low” and “High” indicating the log-rank *p*-value.

Supplementary Figure S2. Association of age of diagnosis and sex associated with immune cell load in glioblastoma

A

COHORT	Age of diagnosis	*P*-value	Sex % Women/Men	*P*-value
**TCGA**				
**High**	61.0 (14.6)	0.535	33.7/66.3	0.774
**Low**	59.9 (20.6)		35.9/64.1	
**Cohort #1**				
**High**	58.0 (13.5)	0.316	37.5/62.5	0.909
**Low**	52.0 (17.5)		40.0/60.0	
**Cohort #2**				
**High**	64.0 (20.0)	0.250	36.9/63.1	0.243
**Low**	60.5 (17.0)		47.9/52.1	

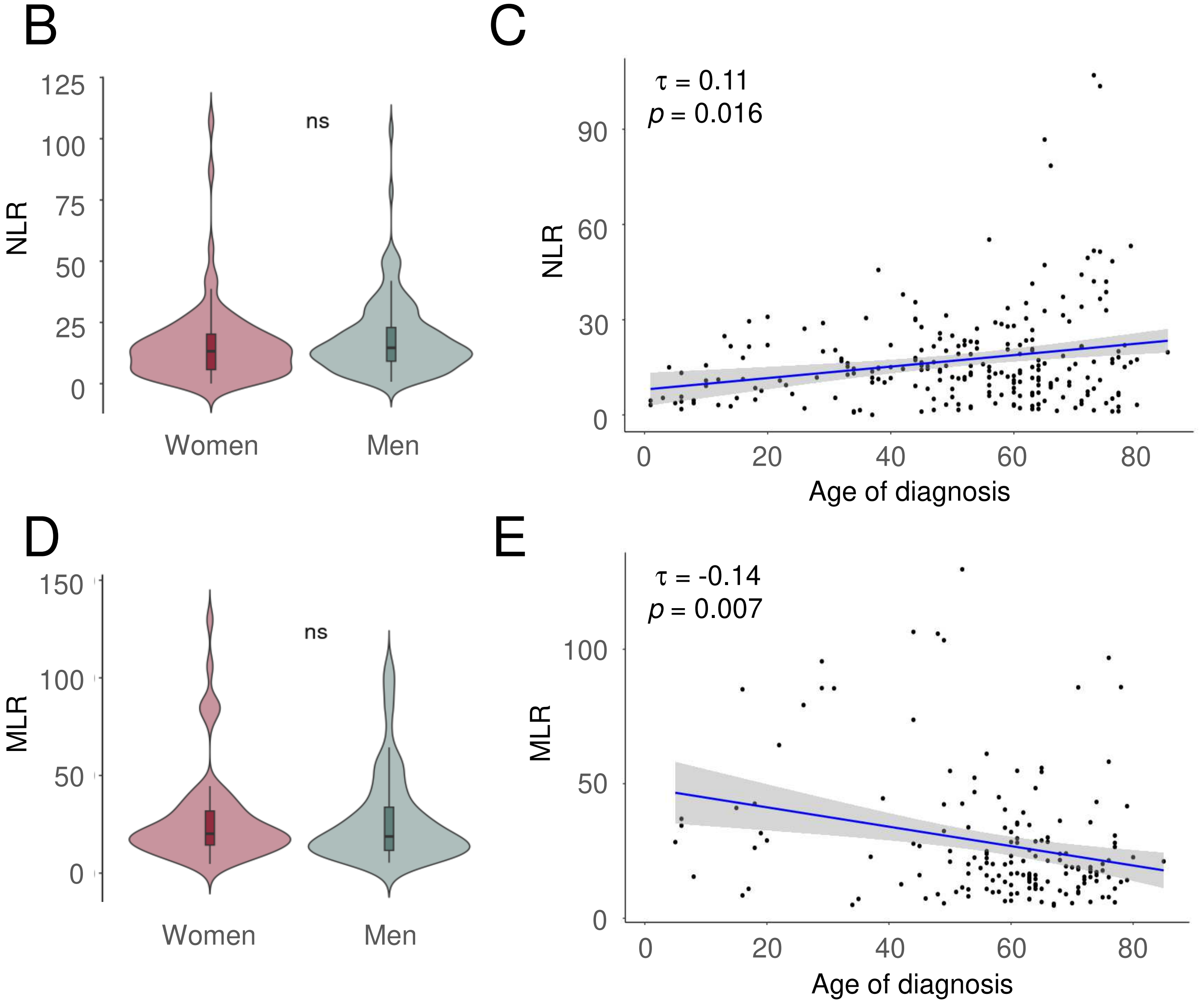

A, Age represented as median (IQR) and sex as % of women/men; *p*-values are obtained from Mann-Whitney *U*-tests. *B-C*, NLR values in the Extended Cohort #2 across sexes (*B*) and age (*C*). *D-E*, the same as B-C for MLR values. ns, not significant.

Supplementary Figure S3. Complete results of the GO analysis in glioblastomas with the most extremes macrophage scores.

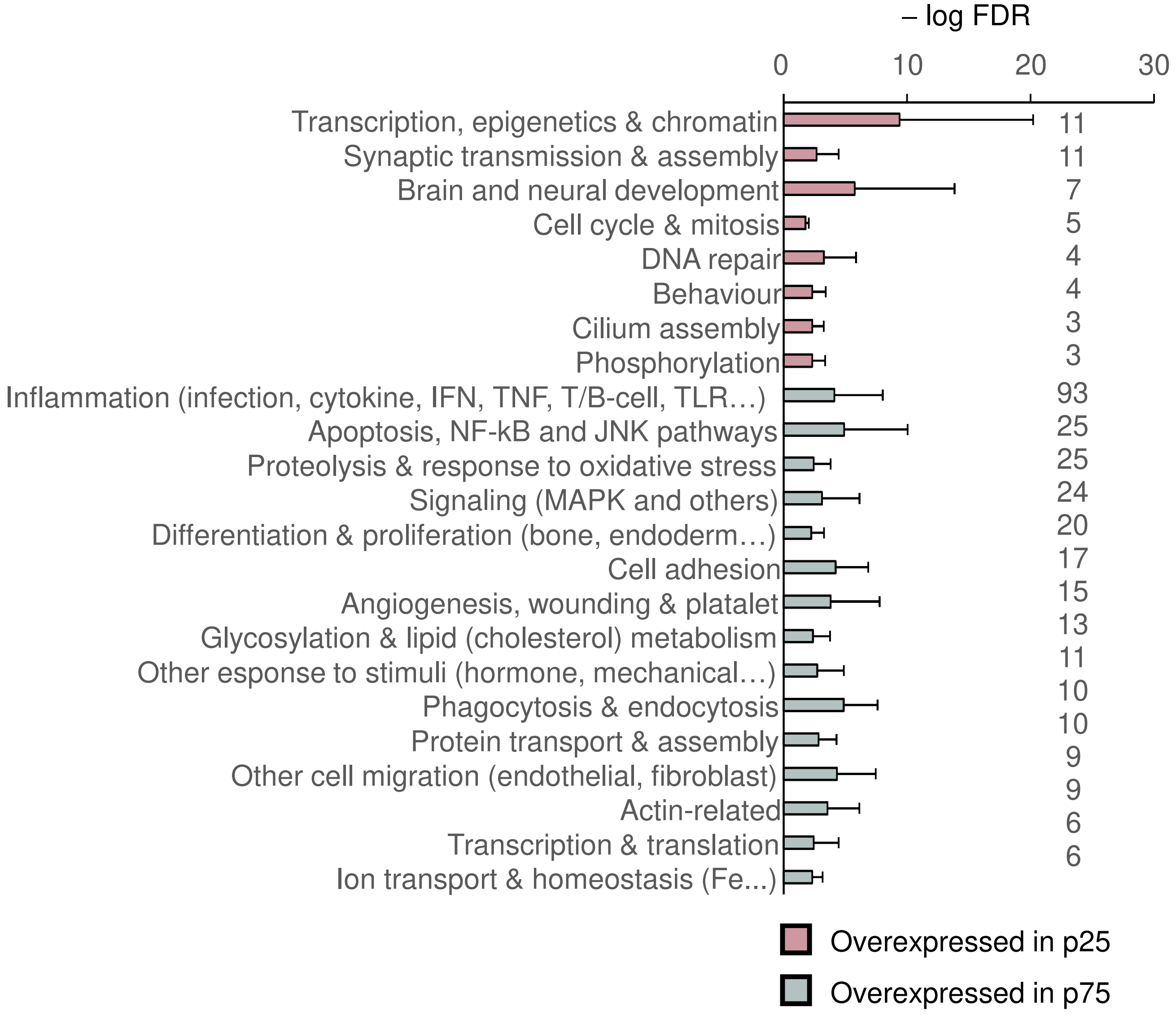

Enrichment analysis of GO terms related with Biological Processes in the differentially expressed genes (adj. *p*-value < 0.05) between glioblastomas with lowest (p25) and highest (p75) macrophage scores. GO terms with FDR < 0.05 were manually grouped in the categories shown in the plot. Data are expressed as mean ± SD, with the corresponding number of GO terms in this category (right to each bar).
